# Cadmium Alters the Biotransformation of Carcinogenic Aromatic Amines by Arylamine *N*-Acetyltransferase Xenobiotic-Metabolizing Enzymes: Molecular, Cellular, and *in Vivo* Studies

**DOI:** 10.1289/ehp.1002334

**Published:** 2010-09-01

**Authors:** Nilusha Ragunathan, Julien Dairou, Elodie Sanfins, Florent Busi, Christophe Noll, Nathalie Janel, Jean-Marie Dupret, Fernando Rodrigues-Lima

**Affiliations:** Université Paris Diderot-Paris 7, Unité de Biologie Fonctionnelle et Adaptative, Centre National de la Recherche Scientifique, Paris, France

**Keywords:** aromatic amine carcinogens, arylamine *N*-acetyltransferases, cadmium, irreversible inhibition, xenobiotic metabolism

## Abstract

**Background:**

Cadmium (Cd) is a carcinogenic heavy metal of environmental concern. Exposure to both Cd and carcinogenic organic compounds, such as polycyclic aromatic hydrocarbons or aromatic amines (AAs), is a common environmental problem. Human arylamine *N*-acetyltransferases (NATs) are xenobiotic-metabolizing enzymes that play a key role in the biotransformation of AA carcinogens. Changes in NAT activity have long been associated with variations in susceptibility to different cancers in relation with exposure to certain AAs.

**Objective:**

We explored the possible interactions between Cd and the NAT-dependent biotransformation of carcinogenic AAs.

**Methods:**

We exposed purified enzymes, lung epithelial cells, and mouse models to Cd and subsequently analyzed NAT-dependent metabolism of AAs.

**Results:**

We found that Cd, at biologically relevant concentrations, impairs the NAT-dependent acetylation of carcinogenic AAs such as 2-aminofluorene (2-AF) in lung epithelial cells. NAT activity was strongly impaired in the tissues of mice exposed to Cd. Accordingly, mice exposed to Cd and 2-AF displayed altered *in vivo* toxicokinetics with a significant decrease (~ 50%) in acetylated 2-AF in plasma. We found that human NAT1 was rapidly and irreversibly inhibited by Cd [median inhibitory concentration (IC_50_) ≈ 55 nM; rate inhibition constant (*k*_inact_) = 5 × 10^4^ M^−1^ · sec^−1^], with results of acetyl coenzyme A (acetyl-CoA) protection assays indicating that Cd-mediated inhibition was due to the reaction of metal with the active-site cysteine residue of the enzyme. We found similar results for human NAT2, although this isoform was less sensitive to inactivation (IC_50_ ≈ 1 μM; *k*_inact_ = 1 × 10^4^ M^−1^ · sec^−1^).

**Conclusions:**

Our data suggest that Cd can alter the metabolism of carcinogenic AAs through the impairment of the NAT-dependent pathway, which may have important toxicological consequences.

Cadmium (Cd), a ubiquitous toxic element and widespread pollutant, is introduced to the environment mainly through anthropogenic activities, such as lead mining, fossil fuel combustion, and the manufacturing of Cd-containing products. Another major source for human exposure to Cd is cigarette smoke (Martelli et al. 2006). The chief route of Cd exposure is via the respiratory system (Potts et al. 2001).

Because of its stability in the environment and long retention time in the human body (half-life, ~ 20 years), Cd can accumulate and cause a variety of adverse effects (Joseph 2009; Waalkes 2003). The target organs for Cd toxicity include the liver, kidney, lung, testis, prostate, and bladder. However, prolonged human exposure to Cd results mainly in diseases affecting lungs and kidneys [International Agency for Research on Cancer (IARC) 1993]. Cd has been classified as a class 1 human carcinogen by IARC (1993). Most of our current knowledge regarding the mechanisms of Cd carcinogenesis is derived from experiments conducted with cell cultures and animal models. These studies have shown that the mechanisms underlying Cd carcinogenesis are multifactorial (Huff et al. 2007). Among these mechanisms, accumulation of DNA damage due to inhibition of DNA repair enzymes by Cd is considered as one of the major underlying processes (Jin et al. 2003; McNeill et al. 2004; Potts et al. 2001). Simultaneous and/or sequential exposure to Cd has been suggested to contribute to the carcinogenic potential of other genotoxic chemicals commonly found in the environment and in the workplace, such as aromatic carcinogens (Prakash et al. 2000; Rivedal and Sanner 1981; Williams et al. 1984).

Aromatic amines (AAs) represent one of the most important classes of occupational or environmental pollutants (Kim and Guengerich 2005). AAs account for 12% of the chemicals known or strongly suspected to be carcinogenic in humans (National Toxicology Program 2005). AAs are by-products of chemical manufacturing (e.g., pesticides, dyestuff, rubbers), gasoline combustion, or pyrolysis reactions (Palmiotto et al. 2001). Carcinogenic AAs such as 4-aminobiphenyl (4-ABP) and β-naphthylamine are also present in cigarette smoke (Hein 1988; Hein et al. 2000).

Human arylamine *N*-acetyltransferases (NATs) are xenobiotic-metabolizing enzymes (XMEs) that play a major role in the biotransformation of AA carcinogens (Dupret and Rodrigues-Lima 2005; Hein 1988). The interrelationship between variable NAT activities and the toxicity or carcinogenicity of environmental AAs has been reported (Badawi et al. 1995; Gemignani et al. 2007; Hein 1988; Minchin et al. 1993). NAT-dependent acetylation of AAs leads mainly to xenobiotic detoxication but also to bioactivation. Detoxification into innocuous compounds is mainly mediated by NAT-dependent *N*-acetylation, whereas *O*-acetylation of chemicals previously hydroxylated by cytochrome P450 1A1 (CYP1A1) or CYP1A2 promotes their metabolic activation into DNA-binding electrophiles (Hein 1988). Several studies have shown associations between NAT1 and/or NAT2 activities and increased risk of cancer, particularly in relation with exposures to AAs (Hein 1988; Hein et al. 2000).

Exposure to both Cd and AAs occurs commonly, such as through cigarette smoke (Joseph 2009; Stavrides 2006). Although Cd has been shown to modify the expression and/or activity of CYP1A1 (Elbekai and El-Kadi 2007; Vakharia et al. 2001), the effect of this metal on the metabolism of aromatic carcinogens remains poorly defined. In this study, we found molecular, cellular, and *in vivo* evidence that Cd, at biologically relevant concentrations, can alter the biotransformation of AA carcinogens through the impairment of the NAT-dependent acetylation pathway. This process may represent an additional mechanism contributing to Cd carcinogenesis.

## Materials and Methods

### Materials

Cadmium chloride (CdCl_2_), 2-aminofluorene (2-AF), 4-ABP, acetyl coenzyme A (acetyl-CoA), coenzyme A (CoA), and protease inhibitors were obtained from Sigma (St-Quentin Fallavier, France). Cell culture reagents were from Invitrogen (Cergy, France). All other reagents were purchased from Euromedex (Soufferweyersheim, France) unless otherwise stated.

### Recombinant enzyme production and purification

Hexa-histidine (6xHis)-tagged human NAT1 was produced and purified from *Escherichia coli* strain BL21 (DE3) as previously described (Dairou et al. 2003). We used the same approach to prepare human NAT2, except that induction with isopropyl β-d-1-thiogalactopyranoside (500 μM) was performed at 30°C for 8 hr followed by a 12-hr incubation at 4°C.

### Effects of Cd on recombinant human NAT enzymes

We tested the effect of Cd on recombinant human NAT1 and NAT2 by incubating purified enzymes (0.3 μM final concentration) with increasing concentrations of CdCl_2_ in 25 mM Tris-HCl (pH 7.5) for 10 min at 37°C. Recombinant NAT1 or NAT2 enzymatic activities were determined spectrophotometrically using *p*-nitrophenylacetate (PNPA) as the acetyl donor and 2-AF as arylamine substrate, as reported previously (Dairou et al. 2003; Mushtaq et al. 2002). In all reaction mixtures, the final concentrations of NAT1 and NAT2 were 15 nM and 30 nM, respectively. For the controls, we omitted the enzyme, 2-aminofluorene (2-AF), or PNPA. All enzyme reactions were performed in triplicate, in conditions in which the initial reaction rates were linear.

We assessed the reversibility of the reaction of Cd with NAT1 and NAT2 by incubating recombinant enzymes with Cd (final concentrations, 0.3 μM for NAT1 and 2 μM for NAT2) for 10 min at 37°C. Mixtures were then dialyzed overnight at 4°C against 25 mM Tris-HCl, pH 7.5. For controls, enzymes not treated with Cd were dialyzed overnight. After dialysis, residual enzyme activities were assayed.

We tested whether reducing agents [reduced glutathione (GSH) and dithiothreitol (DTT)] or the chelating agent EDTA was able to restore the activity of Cd-inhibited NAT1 and NAT2. To this end, recombinant enzymes were preincubated with Cd (final concentrations, 0.3 μM in experiments with NAT1 and 2 μM in those with NAT2) for 10 min at 37°C. Mixtures were then incubated with different concentrations of DTT or GSH (up to 10 mM final concentration) or EDTA (up to 5 mM final concentration) for 10 min at 37°C. Residual enzyme activities were then assessed. Control assays were carried out as described above in the absence of Cd but with GSH, DTT, or EDTA. We also tested whether these reducing or chelating agents were able to protect NAT1 and NAT2 enzymes from the inhibitory effects of Cd by carrying out Cd treatments (final concentration, 0.3 μM in experiments with NAT1 and 2 μM in those with NAT2) in the presence of high concentrations of DTT, GSH, or EDTA (up to 10 mM, which corresponds to > 33,000 times the Cd concentration) and then determining residual enzyme activities.

We investigated the extent to which acetyl-CoA and CoA protected NAT1 and NAT2 from Cd-dependent inhibition. To this end, recombinant enzymes were preincubated with different concentrations of acetyl-CoA or CoA in 25 mM Tris-HCl, pH 7.5, for 5 min at 37°C. Mixtures were then incubated with Cd (final concentration, 0.3 μM for NAT1 experiments and 2 μM for NAT2 experiments) for 10 min at 37°C (final concentration of acetyl-CoA or CoA, 0–3 mM). Samples were then assayed. Control assays carried out in the absence of Cd treatment gave 100% enzyme activity.

### Kinetic analysis: determination of the second-order rate inhibition constant (k_inact_)

NAT1 or NAT2 was incubated under second-order conditions as described previously by Cornish-Bowden (2001). Briefly, recombinant enzymes (0.3 μM final concentration) were incubated with Cd (0.3 μM final concentration) in 25 mM Tris-HCl, pH 7.5, at 37°C. Every 2 min, aliquots of the reaction mixture were taken and quenched by dilution with buffer containing 1 mM EDTA, and the residual enzyme activity was assayed as described below. The values of *k*_inact_ were obtained by fitting residual enzyme activity to the equation


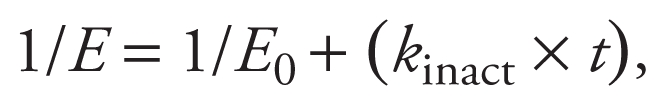


where *E* is the enzyme concentration, *E*_0_ is the initial enzyme concentration, and *t* is time. We used KaleidaGraph, version 3.5 (Abelbeck/Synergy, Reading, PA, USA) for mathematical analyses of the data.

### Cell culture, exposure to Cd, and whole-cell extracts

The murine mtCC1-2 Clara lung epithelial cell line (Magdaleno et al. 1997) was provided by J.M. Sallenave (Institut Pasteur, Paris, France) and grown in Dulbecco’s modified Eagle’s medium (DMEM) supplemented with 20% (vol/vol) fetal bovine serum. Cells were cultured as monolayers in 35- or 100-mm Petri dishes at 37°C. This lung epithelial cell line is known to express only functional Nat2 (the murine ortholog of human NAT1) (Dairou et al. 2009).

Cell monolayers (~ 80% confluence) were washed with phosphate-buffered saline (PBS) and exposed to different concentrations of CdCl_2_ in 10 mL PBS or DMEM for 2 hr at 37°C in a cell incubator. Control cells were incubated with PBS or DMEM only. After incubations, monolayers were washed with PBS and scraped into 0.5–1 mL lysis buffer (25 mM Tris-HCl, pH 7.5; 0.1% Triton X-100) and protease inhibitors. Extracts were sonicated and centrifuged for 15 min at 13,000 × *g*. Supernatants (whole-cell extracts) were removed, and their protein concentration was determined using Bradford reagent with bovine serum albumin as a standard. All cell extracts were adjusted to the same protein concentration by adding lysis buffer and were used for enzyme assays. We determined NAT activity in cells or mouse tissue extracts by measuring the formation of *N*-acetylated metabolites using reverse-phase high-performance liquid chromatography (HPLC) as described previously (Grant et al. 1991). All assays were performed in triplicate under initial reaction rate conditions. Enzyme activities were normalized according to the protein concentration of cellular extracts determined using the Bio-Rad protein assay kit (Bio-Rad, Hercules, CA, USA).

We analyzed CdCl_2_ cytotoxicity using a 3-(4,5-dimethylthiazol-2-yl)-2,5-diphenyltetrazolium bromide (MTT)-based assay (Mosmann 1983) with concentrations of CdCl_2_ ranging from 0 to 100 μM. In the conditions used above, CdCl_2_ cytotoxicity was < 5% for concentrations up to 50 μM.

### Acetylation of AA carcinogens by intact cultured cells in the presence or absence of Cd

Acetylation of 2-AF and 4-ABP by endogenous Nat2 in growing cells was measured by reverse-phase HPLC as described previously (Wu et al. 2000). Cells were incubated with Cd at different concentrations (up to 50 μM) for 2 hr. After treatment, cells were grown in fresh culture medium containing 750 μM 2-AF or 4-ABP. Controls were incubated in the same conditions but with cell monolayers not exposed to Cd. Reactions were found to be linear with time.

In a second set of experiments, Clara cells were coexposed to Cd at different concentrations (up to 50 μM final) and to 750 μM 2-AF (or 4-ABP) in culture medium. At different time points, aliquots were analyzed as described above.

### Mouse Cd exposure and plasma pharmacokinetics

All procedures involving animals were carried out in accordance with the French Agriculture Ministry’s internal guidelines for animal handling. The number of mice and suffering were minimized whenever possible.

We investigated the ability of Cd to inhibit NAT functions *in vivo* by treating 12-week-old female C57BL/6J mice with a sublethal dose of CdCl_2_ (2 mg/kg in PBS), as reported previously (Martin et al. 2007). Two hours after intraperitoneal (IP) injection, mice (*n* = 7) were sacrificed by cervical dislocation, and endogenous NAT activity was measured in protein lysates of tissues known to be targeted by Cd, such as blood, liver, kidney, and lung. Tissue extracts were prepared as described previously (Smelt et al. 2000).

In a second set of experiments, we analyzed the *in vivo* pharmacokinetics of acetylated 2-AF in mice (*n* = 7) with or without CdCl_2_ treatment (2 mg/kg in PBS). Two hours after Cd treatment, 2-AF (50 mg/kg) dissolved in dimethyl sulfoxide was administered by IP injection. Animals were then anesthetized with 10 mg/kg ketamine and 1 mg/kg xylazine, and blood samples were drawn from retro-orbital venous plexus at five sequential time points (30 min, 1 hr, 2 hr, 3 hr, and 6 hr). Samples were diluted 1:50 in HPLC mobile phase and analyzed for parent and acetylated metabolites by HPLC as described above. Area under the curve (AUC) was determined by the trapezoidal rule using Microsoft Excel 2007 (Microsoft Corporation, Paris, France).

### Statistical analysis

Data are presented as mean ± SD of three independent experiments performed in triplicate, unless otherwise stated. One-way analysis of variance was performed, followed by Student’s *t*-test between two groups using StatView 5.0 (SAS Institute Inc., Cary, NC, USA).

## Results

### Cd impairs recombinant human NAT1 and NAT2 activity

**Figure 1:**
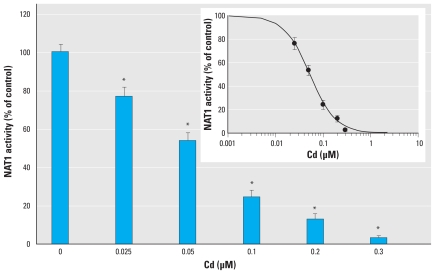
Inhibition of human NAT1 by CdCl_2_. Residual activity after preincubation of NAT1 with increasing concentrations of CdCl_2_ for 10 min at 37°C. Inset: semilogarithm plot of data. Data are presented as mean ± SD as a percentage of control of experiments performed in triplicate, with NAT1 activity determined in triplicate. *p < 0.05 compared with NAT1 activity in the control.

To test whether Cd can inhibit human NAT1 and NAT2 activity, we assessed its effect on purified recombinant enzymes. As shown in Figure 1, Cd demonstrated a dose-dependent inhibitory effect on NAT1 activity. Full inhibition of NAT1 was obtained with Cd concentrations as low as 0.3 μM. The median inhibitory concentration (IC_50_) for Cd was approximately 0.055 μM (Figure 1, inset). We also observed dose-dependent inhibition of the human NAT2 isoform by Cd (data not shown) but with a higher IC_50_ (~ 1 μM). These data indicate that both human NAT isoforms are readily inhibited *in vitro* by low, biologically relevant concentrations of Cd.

### Inhibition of human NAT enzymes by Cd is rapid and irreversible

**Figure 2:**
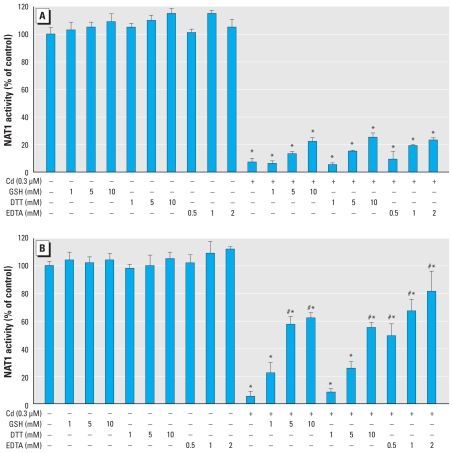
Scavenging effects of GSH and DTT toward Cd-dependent inhibition of NAT1. NAT1 was preincubated with 0.3 μM CdCl_2_ for 10 min at 37°C before (*A*) or in the presence of (*B*) different concentrations of GSH, DTT, or EDTA. The data are mean ± SD of experiments done in triplicate, with NAT1 activity determined in triplicate. *p < 0.05 compared with NAT1 activity in controls. ^#^*p* < 0.05 compared with Cd-inhibited NAT1.

We tested whether the inhibition of NAT1 by Cd could be reversed by physiological (GSH) and nonphysiological (DTT) reducing agents that are known to react with Cd. DTT and GSH (1–10 mM final concentrations) did not significantly reverse Cd-dependent inhibition of NAT1 (Figure 2A). We observed a modest reactivation effect (~ 20% of control activity) with high concentrations of DTT or GSH (10 mM final concentration). Similar results were obtained for human NAT2 (data not shown).

We also tested whether EDTA, a known Cd-chelating agent, was able to reverse Cd-dependent inhibition of NAT1 and NAT2. Incubation of inhibited enzymes with different concentrations of EDTA for 30 min at 37°C did not reverse the inhibitory effect of Cd (Figure 2A). To further analyze the irreversible reaction of Cd with human NAT enzymes, we carried out dialysis experiments. In agreement with the results reported above, dialysis of Cd-inhibited NAT1 and NAT2 enzymes did not allow any significant recovery of enzymatic activity (data not shown). Taken together, these results indicate that Cd-dependent inhibition of NAT enzymes is irreversible.

In a second set of experiments, we analyzed the ability of DTT and GSH to prevent Cd-dependent inhibition of NAT1. Incubation of the enzyme with Cd in the presence of these compounds at high concentrations (> 33,000 times the Cd concentration) protected NAT1 only partially against inhibition (Figure 2B). At high final concentrations of 10 mM GSH or DTT, we observed approximately 60% residual NAT1 activity. Similar results were obtained for NAT2 (data not shown). These data indicate that reducing agents, even at high concentrations, provide only partial protection against Cd-dependent inhibition of NAT enzymes, suggesting that Cd reacts more quickly with NAT enzymes than with GSH or DTT. Conversely, EDTA (2 mM final concentration) provided almost full protection (~ 85%), indicating the dependence of inhibition on the presence of free metal ions in solution (Figure 2B).

**Figure 3:**
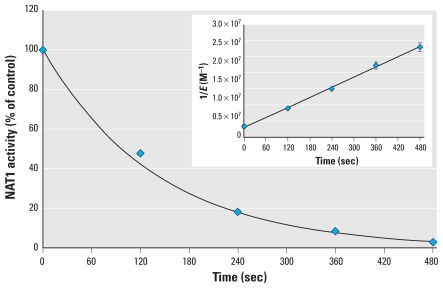
Determination of *k*_inact_ for inhibition of NAT1 by Cd under second-order conditions. After equimolar concentrations of NAT1 and CdCl_2_ were incubated, residual NAT1 activity was assayed at 2-min intervals. Inset: *k*_inact_ obtained by fitting the data to the equation 1/*E* = 1/*E*_0_ + *k*_inact_ × *t* and taking *k*_inact_ from the slope of 1/*E* versus time.

To further characterize the reaction of Cd with NAT enzymes, we performed kinetic analyses. Pseudo-first-order conditions (i.e., implying Cd concentrations were well above enzyme concentration) could not be used because the enzymes were almost instantly inhibited by Cd under these conditions. Therefore, we used second-order conditions (see “Materials and Methods”) to determine the second-order rate constant of inhibition (*k*_inact_). The *k*_inact_ constant for Cd-dependent inhibition of NAT1 was 5.2 × 10^4^ M^−1^ · s^−1^ (Figure 3, inset). The *k*_inact_ for NAT2 was slightly lower (1 × 10^4^ M^−1^ · s^−1^). These results confirm the high reactivity of Cd toward NAT1 and NAT2 enzymes *in vitro*.

### Cd-dependent inhibition is due to interaction with the active-site cysteine residue of NAT enzymes

**Figure 4:**
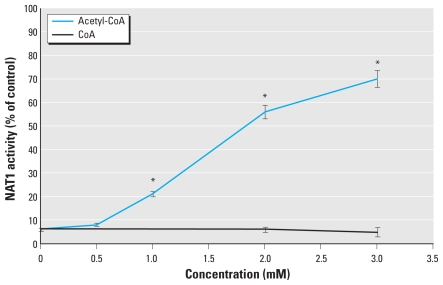
Effect of acetyl-CoA on the Cd-dependent inhibition of NAT1. NAT1 (0.3 μM) was incubated with CdCl_2_ (0.3 μM) in the presence of different concentrations of acetyl-CoA or CoA, and NAT1 activity was determined. The data are mean ± SD of experiments done in triplicate, with NAT1 activity was determined in triplicate. *p < 0.05 compared with Cd-inhibited NAT1.

Alteration of cellular functions by binding to certain thiol groups of biomolecules is the most commonly invoked pathway for Cd toxicity (Joseph 2009). To investigate whether the Cd-dependent inhibition of these XMEs could be due to direct reaction of Cd with the active-site cysteine residue of NAT enzymes, we carried out substrate protection assays using acetyl-CoA and CoA as reported previously (Liu et al. 2008). This protection assay relies on the specific acetylation of the NAT active-site cysteine residue by acetyl-CoA, which protects this residue from further chemical reaction (Liu et al. 2008). Conversely, because CoA is unable to acetylate the active-site cysteine residue, this amino acid is thus susceptible to chemical reaction in the presence of CoA. Acetyl-CoA afforded significant dose-dependent protection against Cd-dependent NAT1 inhibition (up to 70% residual NAT1 activity; Figure 4). In contrast, CoA did not provide any significant protection against Cd-dependent inhibition. We observed similar results with the NAT2 isoform (data not shown). These data suggest that *in vitro*, Cd irreversibly inhibits NAT1 and NAT2 enzymes through the direct interaction with their active-site cysteine residues.

### Impairment of the endogenous NAT-dependent biotransformation pathway in lung epithelial Clara cells by Cd

**Figure 5:**
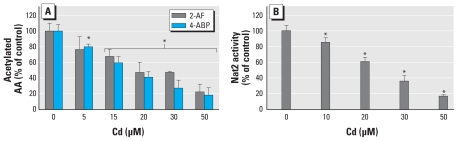
Inhibition of endogenous Nat2 activity and AA acetylation in Clara cells by Cd. Cells in Petri dishes were exposed to different concentrations of CdCl_2_ for 2 hr and then grown in fresh culture medium in the presence of 750 μM 2-AF or 4-ABP. (*A*) The amount of acetylated 2-AF or acetylated 4-ABP quantitated (in triplicate) in culture medium by HPLC. (*B*) Cells were washed, and a whole-cell extract was made; Nat2 activity was measured by HPLC in normalized extracts using 2-AF as substrate. Data are mean ± SD. *p < 0.05.

We tested the effect of Cd on endogenous NAT activity by measuring acetylated metabolites of 2-AF and 4-ABP in the culture medium of mtCC1-2 Clara cells not exposed or previously exposed to different concentrations of Cd. Clara cells are known to play a major role in lung xenobiotic metabolism and are the progenitor cells for bronchogenic carcinomas (Oreffo et al. 1990). Recently, Clara cells were shown to biotransform AA chemicals through the Nat2-dependent pathway (Dairou et al. 2009). We exposed mtCC1-2 Clara cells [which express only Nat2, the murine counterpart of human NAT1 (Kawamura et al. 2008)] to Cd for 2 hr and then to 2-AF or 4-ABP (in fresh medium with no Cd). We observed that the amount of acetylated 2-AF and 4-ABP in cell culture medium decreased in a dose-dependent manner with an IC_50_ value for CD around 17 μM (Figure 5A). We observed similar results in cells coexposed to Cd and AAs (data not shown). Moreover, these results are in agreement with enzyme assays carried out with extracts of treated mtCC1-2 cells (Figure 5B). Overall, these data indicate that exposure to Cd alters the acetylation of AAs in lung epithelial Clara cells through impairment of the endogenous NAT-dependent pathway.

### Effect of Cd on the in vivo NAT-dependent biotransformation of 2-AF

**Figure 6:**
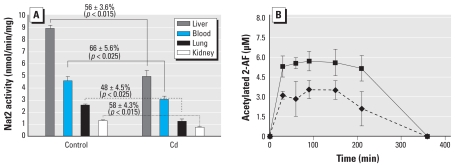
Impairment of Nat2 activity in tissues of mice exposed to Cd (*A*) and pharmacokinetics of acetylated 2-AF (*B*). CdCl_2_ (2 mg/kg) was injected IP. Adult C57BL/6J mice (*n* = 7) were injected IP with 2 mg/kg CdCl_2_. (*A*) Two hours after injection, mice were sacrificed, and Nat2 activity was assessed in lysates from the liver, kidney, lung, and blood. (*B*) Two hours after CdCl_2_ injection, 2-AF (50 mg/kg) dissolved in dimethyl sulfoxide was administered by IP injection, and acetylated 2-AF was measured in blood at different time points. Acetylated 2-AF AUC values: untreated, 1.43 ± 0.15 mmol-min/L; Cd treated, 0.81 ± 0.12 mmol-min/L. Data are mean ± SD.

In C57BL/6J mice (*n* = 7) treated with a sublethal dose of CdCl_2_ (2 mg/kg) as described previously (Martin et al. 2007), we found that Cd to significantly decreased NAT activity in the lungs, kidneys, liver, and blood of treated mice (up to 52% inhibition in the lung; Figure 6A). These data were further confirmed by plasma toxicokinetics experiments with 2-AF, which showed that Cd-treated mice exhibited altered levels of *N*-acetylated 2-AF with an approximately 50% decrease in the AUC (mean ± SD, 1.43 ± 0.15 mmol-min/L for control mice and 0.81 ± 0.12 mmol-min/L for treated mice; Figure 6B). Taken together, these data indicate that Cd alters the NAT-dependent acetylation of 2-AF *in vivo*.

## Discussion

The cellular effects of the toxic metal Cd are manifold. In particular, exposure to Cd is associated with cancers of the prostate, bladder, kidney, and lung (Huff et al. 2007; Waalkes 2003). Because Cd interacts with cellular components in many ways, no factor fully accounts for its spectrum of toxic and carcinogenic effects (Joseph 2009). Several studies have demonstrated that Cd is a complex carcinogen, and the mechanisms underlying Cd carcinogenesis are multifactorial (reviewed by Joseph 2009). Cd interacts synergistically with DNA-damaging agents, such as aromatic carcinogens, which may enhance their mutagenic potential and result in biologically relevant genotoxic effects (Godschalk et al. 2005; Prakash et al. 2000; Rivedal and Sanner 1981; Williams et al. 1984). Although Cd has been suggested to modify the expression of certain XMEs such as CYP1A1 (Elbekai and El-Kadi 2007; Maier et al. 2000), the potential of Cd to alter the biotransformation of aromatic carcinogens remains poorly defined.

NATs are XMEs that play a major role in the biotransformation of AA carcinogens, and changes in the *N*- and/or *O*-acetylation of these chemicals have been linked to carcinogenesis (Hein 1988). We report here that biologically relevant levels of Cd (≤ 50 μM) (Apostolova et al. 2006) alter the biotransformation of carcinogenic AAs through impairment of NAT enzyme functions. We found that Cd can irreversibly inhibit NAT1 and NAT2 acetylation activities *in vitro* with IC_50_ values as low as 75 nM and 1 μM, respectively. Kinetic analysis of these Cd-dependent inhibitions gave second-order *k*_inact_ values of 5 × 10^4^ M^−1^ · s^−1^ and 1 × 10^4^ M^−1^ · s^−1^ for human NAT1 and NAT2, respectively. Studies on DNA repair enzymes known to be impaired by Cd, such as Ogg1 and polynucleotide kinase, have reported IC_50_ values ranging from 5 to 100 μM (Bravard et al. 2006; McNeill et al. 2004; Whiteside et al. 2010; Zharkov and Rosenquist 2002) and *k*_inact_ values around 5 M^−1^ · s^−1^ (Zharkov and Rosenquist 2002). The data we obtained for NAT enzymes indicate that these XMEs are extremely sensitive to Cd exposure, which leads to their rapid functional impairment. Moreover, our results suggest that *in vitro*, NAT1 is more susceptible to Cd-dependent inhibition than is NAT2. A similar trend with isoform-selective inactivation of human NAT enzymes by 4-nitrosobiphenyl and 2-nitrosofluorene was recently reported, with NAT1 appearing to be more sensitive to these compounds (Liu et al. 2009).

Cd has been reported to act as either a reversible or an irreversible inhibitor of certain enzymes, depending on the nature of the enzyme–Cd interaction. For instance, Whiteside et al. (2010) have shown that different DNA repair enzymes exhibit different behaviors in this regard. In the present study, we found that the *in vitro* inhibition of NAT1 and NAT2 by Cd was irreversible because extensive dialysis did not restore enzyme activities. Moreover, the reducing agents GSH or DTT and the chelating agent EDTA did not restore the activity of NAT1 and NAT2, further supporting the irreversible nature of the Cd-dependent inhibition of these XMEs. We also found that the presence of high concentrations of GSH or DTT (10 mM final) afforded only partial protection (~ 60% residual activity) against Cd-dependent inhibition (with Cd at a molar concentration > 33,000 times lower than that of GSH or DTT). These data suggest that Cd reacts more rapidly with NAT1 (and NAT2) than with GSH or DTT. Accordingly, the kinetics of the Cd reaction with GSH is at least two orders of magnitude lower (*k*_assoc_ < 10^2^ M^−1^ · s^−1^) than the inactivation rates found for NAT1 and NAT2 (*k*_inact_ > 10^4^ M^−1^ · s^−1^). Conversely, we found that EDTA afforded significant protection against Cd-dependent inhibition of human NAT enzymes. These results are similar to data obtained with the DNA repair enzyme Ogg1 and indicate the dependence of inhibition on the presence of free metal ions in solution (Zharkov and Rosenquist 2002). Acetyl-CoA protection assays indicated that Cd-dependent irreversible inhibition of human NAT1 and NAT2 was due to the reaction of the metal with the reactive catalytic cysteine residue (Ragunathan et al. 2008), which is in agreement with the fact that Cd exhibits high affinity for certain reactive thiols (Bravard et al. 2006). A similar mechanism of inhibition has been reported for other enzymes inhibited by Cd, such as Ogg1 and nicotinamide adenine dinucleotide phosphate–dependent isocitrate dehydrogenase (Kil et al. 2006).

The amount of Cd absorbed in the body after exposure is principally due to inhalation of Cd-contaminated smoke and particles and results mainly in diseases affecting kidneys and lungs (Joseph 2009). Cd has been shown to accumulate in tissues at up to tens-of-micromolar concentrations (Jin et al. 2003). Furthermore, occupational exposures in industrial society have been reported to produce renal cortical Cd concentrations around 300 μM (Apostolova et al. 2006). We exposed murine Clara cells, which are known to express functional Nat2, the murine ortholog of human NAT1 (Kawamura et al. 2008; Minchin et al. 2007), to biologically relevant concentrations of Cd (≤ 50 μM). We found that Cd impaired the NAT-dependent biotransformation of two carcinogenic AAs (4-ABP and 2-AF) through inhibition of the endogenous Nat2 enzyme with an IC_50_ close to 20 μM. Similar IC_50_ values have been reported for enzymes such as caspase 3 (Yuan et al. 2000) and apurinic/apyrimidic endonuclease 1 (McNeill et al. 2004) inhibited by Cd in cultured mammalian cells. Moreover, exposure of lung alveolar epithelial cells to Cd has been shown to significantly reduce the activity of the DNA repair enzyme formamidopyrimidine DNA glycosylase (Potts et al. 2001). Our results are also in agreement with previous data showing that the acetylation of 3-chloroaniline in isolated rat hepatocytes could be suppressed by Cd concentrations close to 25 μM (Alary et al. 1989).

Cd significantly inhibited (by 36–52%) endogenous NAT activity in lung, liver, kidney, and blood, tissues known to accumulate Cd (Huff et al. 2007; Joseph 2009), from mice exposed to Cd IP. These data indicate that NAT-dependent biotransformation of AAs may be altered by Cd in several tissues expressing these XMEs. Toxicokinetics studies provided further evidence that *in vivo* Cd alters the NAT-dependent biotransformation of carcinogenic AAs. Mice exposed to Cd exhibited altered biotransformation of 2-AF, as shown by the 50% decrease in acetylated 2-AF AUC.

Increasing evidence suggests that genotoxicity induced by Cd also depends on the synergic interactions of Cd with genotoxic chemicals (Joseph 2009). Cd inhibits several enzymes involved in DNA repair, and this has been identified as a major mechanism underlying the carcinogenic potential of Cd (Joseph 2009; Zharkov and Rosenquist 2002). Furthermore, synergic interactions between Cd and carcinogenic aromatic chemicals that can lead to aromatic DNA adducts have been reported (Godschalk et al. 2005; Prakash et al. 2000; Rivedal and Sanner 1981; Williams et al. 1984). Moreover, studies have shown that Cd can modify the expression and/or activity of certain XMEs, such as CYP1A1, leading to altered metabolism of polycyclic aromatic hydrocarbons such as benzo[*a*]pyrene (Elbekai and El-Kadi 2007; Maier et al. 2000; Vakharia et al. 2001). Interestingly, Cd has been proposed to enhance mutagenicity of benzo[*a*]pyrene metabolites (Prakash et al. 2000).

Cd toxicity is caused by both acute and chronic exposure. Although the toxicity of Cd is considered mostly chronic, several studies using acute conditions have yielded a substantial amount of information pertinent to Cd toxicity, including mechanistic information (Bravard et al. 2006; McNeill et al. 2004). Moreover, acute inhalation of high levels of Cd in humans may result in long-lasting impairment of lung functions (Agency for Toxic Substances and Disease Registry 1997). Here, we showed that acute exposure to Cd alters the biotransformation of AAs in several tissues, including lung. Further studies are needed to assess whether chronic exposure to Cd leads to similar effects.

Humans are exposed to AA carcinogens mainly through cigarette smoke or occupational/industrial pollutants. Several studies have suggested that NAT-dependent acetylation is a susceptibility factor for cancers associated with AA exposures (Hein 1988), possibly through formation of aromatic DNA adducts (Badawi et al. 1995). Interestingly, simultaneous and/or sequential exposure to Cd and AAs occurs commonly, such as in cigarette smoke (Hein et al. 2000; Stavrides 2006). Our results provide evidence that Cd alters AA carcinogen metabolism by interfering with the NAT-dependent acetylation pathway. This may represent an additional mechanism contributing to Cd carcinogenesis.
